# Light smoking at base-line predicts a higher mortality risk to women than to men; evidence from a cohort with long follow-up

**DOI:** 10.1186/1471-2458-14-95

**Published:** 2014-01-30

**Authors:** Margaret A Hurley

**Affiliations:** 1 School of Health, University of Central Lancashire, Preston, UK

## Abstract

**Background:**

There is conflicting evidence as to whether smoking is more harmful to women than to men. The UK Cotton Workers’ Cohort was recruited in the 1960s and contained a high proportion of men and women smokers who were well matched in terms of age, job and length of time in job. The cohort has been followed up for 42 years.

**Methods:**

Mortality in the cohort was analysed using an individual relative survival method and Cox regression. Whether smoking, ascertained at baseline in the 1960s, was more hazardous to women than to men was examined by estimating the relative risk ratio women to men, smokers to never smoked, for light (1–14), medium (15–24), heavy (25+ cigarettes per day) and former smoking.

**Results:**

For all-cause mortality relative risk ratios were 1.35 for light smoking at baseline (95% CI 1.07-1.70), 1.15 for medium smoking (95% CI 0.89-1.49) and 1.00 for heavy smoking (95% CI 0.63-1.61). Relative risk ratios for light smoking at baseline for circulatory system disease was 1.42 (95% CI 1.01 to 1.98) and for respiratory disease was 1.89 (95% CI 0.99 to 3.63). Heights of participants provided no explanation for the gender difference.

**Conclusions:**

Light smoking at baseline was shown to be significantly more hazardous to women than to men but the effect decreased as consumption increased indicating a dose response relationship. Heavy smoking was equally hazardous to both genders. This result may help explain the conflicting evidence seen elsewhere. However gender differences in smoking cessation may provide an alternative explanation.

## Background

A recent cohort study of over one million women born around 1940 in the UK showed that two thirds of deaths of women smokers in their 50s, 60s and 70s were due to smoking related disease and that women smokers lost at least 10 years of life [1]. For the US, it has been estimated that there were 291,000 smoking attributable deaths among men and 229,000 deaths among women in the years 2002 to 2006 [2]. Hence, there is universal agreement that smoking tobacco is harmful to all. However, for over a decade there has been a continuing critical debate as to whether the same level of smoking exposure is more harmful to women than to men [3-11]. A number of studies have shown that the hazard of lung cancer, and similarly the hazard of chronic obstructive pulmonary disease, are about equal for men and for women whereas other studies have demonstrated that women smokers are at greater risk of smoking-related disease, such as coronary heart disease, compared to men for the same amount of smoking [12-14]. A recent comparison of two different Australian cohorts one of men and one of women, concluded that men and women with similar patterns of smoking experience had similar mortality from smoking related disease [15]. Yet, the accompanying editorial concluded that the total evidence to date from both epidemiology and biology suggested that there might well be differences in the health consequences of smoking for men and women but that study limitations obscured the evidence for a gender difference in smoking-related mortality [16]. Previous studies have not used an individual relative survival approach and this certainly has been a limitation of published analyses [17]. An individual relative survival analysis makes the very best use of the available information on an individual and can find subtle differences that aggregated data may fail to detect. Individual relative survival provides a powerful statistical analysis. Also cohorts are rare which contain both men and women together and which have been followed up for many years. This paper uses an individual relative survival approach to the analysis of a cohort of cotton mill workers which contained 42 years of mortality data and looks at the female to male relative risk of smoking compared to never having smoked.

## Methods

### Participants

**Figure 1:**
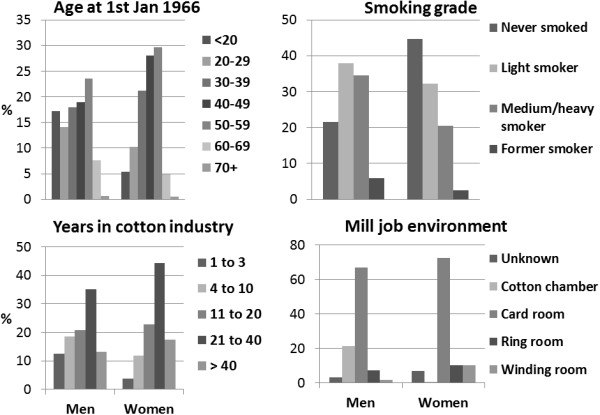
**Worker demographics. **Histograms that show the demographics of the men and women cotton workers.

In 1966 Her Majesty’s Factory Inspectorate (now part of the UK Health and Safety Executive) initiated a study of workers in cotton manufacturing mainly in Lancashire, England. From 1966 to 1970, around 3500 workers from 52 mills were medically examined to determine their current respiratory health. This included a physical lung function test to determine forced expiratory volume in one second (FEV_1_) and forced vital capacity (FVC). A health questionnaire ascertained smoking grade; never smoker, light smoker (1–14), medium smoker (15–24), heavy smoker (≥ 25 cigarettes per day) and former smoker together with presence of cough and phlegm at least 3 days per week for at least 3 months of the year and whether the worker had the lung disease Byssinosis. Height was also recorded. Demographics of the cohort are given in Table 1. The workers were later traced and flagged for vital status at the National Health Service (NHS) Central Register for England and Wales (now the NHS Information Centre) with the aim of exploring the effect of current respiratory health on future longevity and cause of death. By 1972 most mills had closed [18]. Analyses of the mortality to 1984 and to 2007 have been reported [19,20]. By the end of follow-up at 31^st^ December 2007, 2018 workers had died before age 90 years (Table 1). The focus of earlier studies was to ascertain whether exposure to cotton dust was protective for lung cancer [19,20]. The cotton industry in Lancashire was notable in that it provided full-time employment for very many women. Therefore, this cohort contained both men and women working mostly full-time, doing identical jobs in the work place and in the same work place settings and with a high proportion of women smokers across the spectrum of consumption (Figure 1). Men and women’s ages spanned the entire age range from less than 20 years to more than 70 years and men and women both had a similar employment history in terms of years in the industry (Figure 1). Hence these data on men and women are well matched and provide a serendipitous opportunity for making gender comparisons of the influence of smoking on the subsequent mortality of the men and women workers.

**Table 1:** **Participants in the cohort at recruitment and follow-up to 31**^**st **^**December 2007**

	**Men N = 1548**	**Women N = 1911**
**Demographics at recruitment:**		
Age at 1 Jan 1966 (yrs): Mean (SD)	39.2 (15.6)	43.1 (12.3)
Age at 1^st^ examination (yrs): Mean (SD)	41.5 (15.6)	45.6 (12.2)
Time worked in cotton industry (yrs): 1 to 3	193 (13%)	73 (4%)
4 to 10	286 (19%)	226 (12%)
11 to 20	322 (21%)	435 (23%)
21 to 40	542 (35%)	846 (44%)
More than 40	205 (13%)	331 (17%)
Smoking status: Never smoked	333 (22%)	856 (45%)
Light smoker (1–14 cigarettes per day)	589 (38%)	615 (32%)
Medium smoker (15–24 cigarettes per day)	445 (29%)	337 (18%)
Heavy smoker (25+ cigarettes per day)	91 (6%)	54 (3%)
Former smoker	90 (6%)	49 (3%)
**Follow-up:**		
Number of workers	1548	1911
Embarked before age 90	44	44
Embarked after age 90	0	2
Censored at age 90 then died later	40	101
Censored at age 90 and still alive	6	39
Died before age 90	900	1118
Alive and younger than age 90	558	607

### Statistical methods

An individual relative survival approach was used because age at entry to the cohort in 1966 to 1970 varied widely, from less than 20 to over 70 years of age [21-23]. It was also required because residual life expectancy had rapidly increased in England between 1966 and 2007 [24]. Relative survival is becoming recognised as the most appropriate approach for many cohort studies [25]. Individual relative survival was calculated using mortality at mid-year population estimates for England and Wales for 1966 to 2007 for all-cause mortality and for six specific causes of death, namely lung cancer, all cancers except lung, circulatory systems disease, ischemic heart disease, cerebrovascular disease, respiratory system disease and bronchitis, emphysema and other COPD [26]. The different ICD codes for these disease groupings over the time interval 1966 to 2007 were given previously [20]. Using these reference population estimates, the observed survival times were transformed to the measure of individual relative survival [21]. In a similar way to Poisson regression using expected values, the computation of the measures of individual relative survival adjusts for the effects of age and chronological time at entry, and also increasing age and advancing chronological time during follow-up and is the strength of this approach [21-23,27]. The individual relative survival times were then modelled using Cox’s regression adjusted for the confounding variables; byssinosis, cough and phlegm, time worked in the cotton industry, FEV_1_% predicted and FVC, scaled appropriately to aid interpretation [21-23]. The risk factors of interest were included, namely smoking grade at baseline, gender and the interaction between smoking grade and gender. The interaction was parameterised as three or four female to male, smoker to non-smoker relative risk ratios (RRR). These were for light, medium, heavy and former smoking in the case of four RRRs (all-cause mortality) or light, medium/heavy and former smoking in the case of three RRRs (cause specific and all-cause mortality). The proportional hazards assumption was tested for the Cox’s regression model using the correlation coefficient (*ρ*) between the scaled Schoenfeld residuals and the transformed survival times. Further, for all-cause mortality, the individual relative survival measure (Y) was transformed to Z = -log(1-Y) where Z is an alternative measure of individual relative survival. The alternative measure yields identical model coefficients when analysed using Cox’s regression model, which is semi-parametric, but has the property that it can be modelled fully parametrically using, for example, the Weibull model. Therefore this latter measure provides mortality predictions for different scenarios. The programming was carried out in the R programming language [28].

### Dependent and independent variables

In detail, the dependent variable in the Cox and Weibull regressions was the transformed survival time, the transformation being that which removed the effects of age and chronological time using the gender specific England and Wales mortality rates from 1966 to 2007 [26]. For each participant the measure of individual relative survival was the transformed survival time.

The independent variables in the regression were those ascertained at first medical examination, namely

1. Smoking grade category; never smoker, light smoker (1–14), medium smoker (15–24), heavy smoker (≥ 25 cigarettes per day) and former smoker

2. Gender; male (coded 0) or female (coded 1)

3. Female light smoker; no (coded 0) or yes (coded 1)

4. Female medium smoker; no (coded 0) or yes (coded 1)

5. Female heavy smoker; no (coded 0) or yes (coded 1)

6. Female former smoker; no (coded 0) or yes (coded 1)

Indicator variables 3, 4, 5 and 6 provided estimates of the four RRRs for light, medium, heavy and former smoking and comprise the four degrees of freedom of the gender smoking grade interaction.

7. Presence of the lung condition Byssinosis; no (coded 0) or yes (coded 1)

8. Presence of cough and phlegm; no (coded 0) or yes (coded 1)

9. Lung function covariate expressed as (100 - FEV_1_% predicted)/10. If lung function was 100% of that predicted for a person of that same age, height and gender, then the covariate takes the value zero. If lung function was 90% of that predicted for a person of that same age, height and gender, then the covariate takes the value 1. The hazard ratio in the survival model can then be interpreted as the increased mortality hazard for a 10% reduction in FEV_1_% predicted at time of first medical examination.

10. Lung function covariate expressed as (100(FEV_1_/FVC)-100)/10. If the FEV_1_ to FVC ratio is 1 then the covariate takes the value zero. If FEV_1_ is 90% of FVC (reduced lung function) then the covariate takes the value 1 and the hazard ratio in the survival model can be interpreted as the increased mortality hazard for a 10% reduction in the ratio.

11. Height at first medical examination.

### Ethical approval

Ethical approval for these studies was obtained from the University of Central Lancashire’s Faculty of Health Research Ethics Committee which accepted that the study had been granted exemption by the Department of Health’s National Information Governance Board from the need to obtain informed consent from individuals retrospectively to participate in the mortality study. In addition the Medical Research Information service at the NHS Information Centre granted permission for the study to receive vital status data.

## Results

The correlation coefficient (*ρ*) between the scaled Schoenfeld residuals and the transformed survival times are shown in Table 2 and all correlations were less than 0.05 in magnitude indicating that the proportional hazards assumption was acceptable. The anticipated effect of smoking on all-cause mortality was evident (Table 2) with hazard ratios (HR) in the Cox regression of 1.21 (95% CI 1.00-1.46) for light smoking, 1.68 (95% CI 1.34-2.00) for medium smoking and 1.99 (1.49-2.65) for heavy smoking relative to never having smoked. The female to male RRRs demonstrated a dose response relationship; the RRR was significantly elevated above unity for light smoking (1.35; 95% CI 1.07-1.70) was lower but not significant for medium smoking (1.15; 95% CI 0.89-1.49) and lower again and insignificant for heavy smoking (1.00; 95% CI 0.63-1.61). The parametric Weibull model was found to be an acceptable fit to the alternative measure of individual relative survival with highest likelihood amongst a range of tested alternative models. The HRs and RRRs were virtually identical to those obtained by the Cox model implying robustness to choice of model. It would seem therefore, for this cohort at least, that light smoking at baseline was relatively more harmful to women than to men in terms of all-cause mortality. The effect reduced as smoking level increased and there was no gender difference for heavy smokers.

**Table 2:** **Hazard ratios (HR) for all-cause mortality to 31**^**st **^**December 2007 from the regression models**

			Weibull model^*^				**Cox model**		**Proportional hazards assumption**	
	**HR**		**95% CI**	**P**	**HR**		**95% CI**	**P**	***ρ***	**P**
Never smoked	1.00				1.00					
Light smoker	1.21	1.00	1.46	.051	1.21	1.00	1.46	.055	-0.012	.60
Medium smoker	1.68	1.38	2.05	<.001	1.64	1.34	2.00	<.001	0.000	.99
Heavy smoker	2.01	1.51	2.68	<.001	1.99	1.49	2.65	<.001	-0.042	.06
Former smoker	0.89	0.66	1.21	.465	0.89	0.66	1.22	.475	-0.012	.60
Male	1.00				1.00					
Female	1.10	0.92	1.32	.304	1.07	0.89	1.29	.471	-0.020	.36
Light smoker RRR	1.35	1.07	1.70	.012	1.35	1.07	1.70	.012	-0.008	.71
Medium smoker RRR	1.13	0.87	1.46	.361	1.15	0.89	1.49	.290	-0.029	.19
Heavy smoker RRR	0.98	0.61	1.58	.942	1.00	0.63	1.61	.990	-0.021	.35
Former smoker RRR	1.11	0.68	1.80	.681	1.10	0.68	1.78	.705	-0.026	25
Byssinosis absent	1.00				1.00					
Byssinosis present	1.04	0.93	1.16	.484	1.04	0.93	1.15	.535	-0.010	.67
Cough and phlegm absent	1.00				1.00					
Cough and phlegm present	1.07	0.97	1.18	.158	1.07	0.97	1.18	.155	0.031	.17
One decade in the cotton industry	0.91	0.88	0.94	<.001	0.91	0.88	0.95	<.001	0.047	.04
FEV_1_: 10% decrease below normal	1.07	1.04	1.10	<.001	1.07	1.04	1.10	<.001	0.014	.54
FEV_1_ to FVC ratio: decrease of 10%	1.01	0.95	1.07	.763	1.01	0.96	1.07	.726	0.001	.97

*LogL = -1813.7, Shape 1.08 (95% CI 1.04 to 1.12).

It has been suggested that any gender difference in risk can be explained by the difference in the physical size of women compared to men [29]. The same consumption in a smaller pair of lungs might induce greater damage. Hence the logarithm of height was added to the models, assuming an allometric relationship between height and lung volume. This variable was not even close to significant and the RRR values were unchanged. The gender difference seen in this cohort could, therefore, not be ascribed to size difference.

Since lung function is affected by smoking, the two lung function variables were removed from the model. As a consequence light smoking became more significant (P = 0.01 in the Cox model) but the RRRs remained unchanged. When only the variables gender, smoking grade and the RRRs remained in the model, the RRR for light smoking increased to 1.39 (95% CI 1.10 to 1.75) in the Cox model (P = 0.005). Therefore, the result regarding the greater mortality risk to women of light smoking at baseline in this cohort was robust to the inclusion of confounding variables.

The cause specific female to male RRRs were estimated using the same model as for all-cause mortality, except that three RRRs were estimated since numbers of deaths were smaller than for all-cause mortality. The estimates are shown in Table 3. For light smoking the RRRs are elevated above unity for all specific causes, significantly so for circulatory system diseases (P = 0.042) and close to 5% significance for respiratory system disease (P = 0.054) and bronchitis, emphysema and other COPD (P = 0.052). The estimated RRR for the latter was 3.4 (95% CI 1.0 to 11.9) indicating that, for women, mortality risk may be of the order of three times that for men. For medium/heavy smoking there was no evidence of a greater risk to women compared to men.

**Table 3:** Relative risk ratios (RRR) estimated by Cox’s regression for different causes of death

	**Light smokers**				**Medium/heavy smokers**				**Former smokers**			
	**RRR**	**95% CI**		**P**	**RRR**	**95% CI**		**P**	**RRR**	**95% CI**		**P**
All causes	1.35	1.07	1.70	0.012	1.12	0.87	1.43	0.381	1.10	0.67	1.78	0.705
Lung cancer*	1.40	0.22	9.12	0.724	2.00	0.31	12.6	0.466				
All cancers excluding lung	1.09	0.65	1.85	0.735	1.07	0.60	1.89	0.825	0.77	0.22	2.68	0.685
Circulatory systems disease	1.42	1.01	1.98	0.042	1.05	0.73	1.51	0.802	1.03	0.53	2.02	0.924
Ischemic heart disease	1.33	0.86	2.07	0.203	0.99	0.62	1.59	0.971	1.16	0.46	2.93	0.749
Cerebrovascular disease	1.45	0.74	2.85	0.278	1.09	0.51	2.30	0.829	1.01	0.31	3.33	0.983
Respiratory system disease	1.89	0.99	3.63	0.054	1.58	0.81	3.07	0.182	2.09	0.68	6.41	0.197
Bronchitis, emphysema and other COPD	3.44	0.99	11.9	0.052	2.71	0.78	9.39	0.116	6.68	1.04	42.9	0.045

*No women former smokers died of lung cancer.

Table 4 shows the predicted 5%, 50% (median) and 95% percentile for the residual lifetime for a cohort participant aged 25, 45 and 65 years of age at 1^st^ January 1965. These values were obtained from the Weibull model which included gender, smoking grade and four RRRs. For an individual of 45 years of age, light smoking reduced a man’s median residual lifetime by 2.6 years compared to never having smoked, whereas for a woman the reduction was 5.9 years (3.3 years difference in median years of life lost). For medium smoking the analogous values are 6.4 for men and 7.6 for women (1.2 years difference in reduction) and for heavy smoking 8.2 years for men and 7.8 years for women (-0.4 years difference in reduction). This provides another way of understanding the greater risk to women of light smoking compared to men for this cohort.

**Table 4:** **Residual lifetime percentiles conditional on age at 1**^**st **^**January 1965 and reported smoking status**

		**Age at death (years)**							
		**Never smoked**		**Light smoker**		**Medium smoker**		**Heavy smoker**	
**Predicted survival percentile**^**1**^		**Men**	**Women**	**Men**	**Women**	**Men**	**Women**	**Men**	**Women**
Age at 1^st^ Jan 1965									
25 years	5%	54.92	59.50	52.33	53.33	49.08	51.67	47.08	51.42
	50%	82.83	85.83	80.75	81.33	77.17	80.17	75.50	80.00
	95%	100.50	103.92	96.33	94.08	91.25	92.08	89.17	91.83
45 years	5%	55.25	58.92	54.00	55.00	52.00	53.92	51.17	90.50
	50%	78.50	84.33	75.92	78.42	72.08	76.75	70.33	76.50
	95%	98.00	102.58	93.75	92.75	88.67	90.83	86.58	53.75
65 years	5%	66.75	68.42	66.62	67.00	66.00	66.75	65.83	66.75
	50%	78.58	83.33	76.75	79.08	74.58	77.75	73.33	77.58
	95%	94.00	100.08	90.58	91.25	86.58	89.50	85.17	89.25

^1^assumes force of mortality remains constant at 2007 values from 2008 onwards.

**Figure 2:**
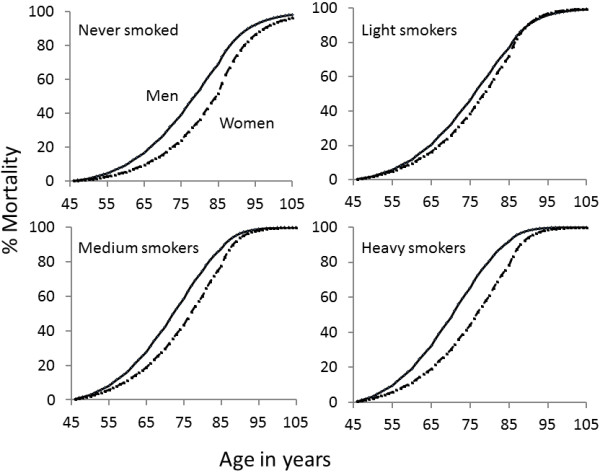
**Mortality curves.** Predicted mortality curves for men and women who attained age 45 at 1^st^ January 1965 and who never smoked, were light smokers (1 to 14 cigarettes per day), medium smokers (15 to 24 cigarettes per day) or heavy smokers (25 or more cigarettes per day). Solid line shows mortality for men and broken line for women.

The predicted mortality percentages for cohort participants aged 45 at 1^st^ January 1965 for men and women who had never smoked, who were light, medium or heavy smokers, are shown in Figure 2. The survival advantage of women over men is clear for those who have never smoked, since the gap is wide. For light smokers the gender gap is considerably narrowed but as smoking grade increases to heavy smoking, the full gender gap becomes re-established. The interaction between smoking grade and gender is clearly visible.

## Discussion

This study shows clear evidence that light smoking measured at baseline predicted a higher mortality risk to women than to men for participants in the cotton workers’ cohort and that an elevated risk was evident for diseases of the circulatory system and the respiratory system, especially bronchitis, emphysema and other COPD. The results of these analyses are in contrast to those obtained from the analysis of two Australian cohorts [15]. The Australian cohorts were large; 12,154 men and 11,707 women with 3549 and 2665 deaths respectively during the 10-year follow-up period. However the Australian cohorts contained only 1317 and 912 current smokers of whom 809 men and 434 women were light smokers. Age at entry to the Australian cohort was 65 for men and 70 for women and so these cohorts contained only those who were elders. The cotton workers cohort was much smaller with 1548 men and 1911 women but 900 and 1118 deaths before age 90. Those recorded as current smokers at baseline were 1125 men and 1006 women of whom 589 and 615 respectively were light smokers. Thus the cotton workers’ cohort had more women smokers and more women light smokers than the Australian cohort despite the latter’s much larger size. The follow-up for the cotton workers’ cohort was for 42 years and age at entry covered the full age range from less than 20 years to over 70 years. Estimates of hazard ratios from the US 1997–2004 National Health Interview Survey and its follow-up showed higher hazard ratios for women compared to men for all four levels of smoking grade in age groups 35 to 44, 45–54 and 55 to 64 years. For the age groups 65 to 74 and 75 to 84 hazard ratios were similar [2]. These results might suggest that a cohort of men and women recruited after age 65 would not give estimates applicable across the full age range. Therefore it is possible that the results from the cotton workers’ cohort may have wider validity than that for the Australian cohorts.

A systematic review and meta-analysis of the risk of coronary heart disease estimated the women-to-men relative risk ratio of smoking compared to not smoking at 1.25 (95% CI 1.12 to 1.39) [14]. The relative risk ratio obtained from the cotton workers’ cohort study for mortality from ischematic heart disease was 1.33 (95% CI 0.86 to 2.07) for light smoking compared to not smoking. Clearly these two relative risk ratios are not quite comparable but they do demonstrate that the magnitudes of estimates obtained in this study were plausible. It has also been estimated previously that acute myocardial infarction occurs earlier in women smokers than in men smokers and twice as many years are lost by women smokers from this cause as by men smokers [30]. These findings accord with the current study for light smokers since the estimated median residual lifetime of a 45 year old woman light smoker was reduced by 5.9 years compared to 2.6 years for her male counterpart in the cohort. A matched case control study using the UK medical research database known as The Health Improvement Network (THIN) demonstrated that smoking carried a significantly higher risk of lung cancer in women compared to men [31]. The estimated relative risk ratio was 1.5 for women ever smoking 20 or more cigarettes per day and this ratio accords with the current study where the estimated ratio was 1.4 for women smoking 1 to 14 cigarettes per day and 2.0 for women smoking 15 or more cigarettes per day at baseline. The THIN study similarly found that height provided no explanation for the gender difference. A systematic review and meta-analysis of 81 cohorts looked at smoking as a risk factor for stroke in women compared to men and concluded that there was a similar risk overall [32]. This concurs with the current study which found no evidence for a gender difference in mortality from cerebrovascular disease.

The present study showed the greatest median number of years of life lost was 8 years for women born around 1920 who were heavy smokers. This estimate is less than that for the million women’s study for which a 10 year loss of life was estimated [1]. The two estimates are not wholly comparable because of the difference in age at recruitment and the stage at which smoking status was recorded but are sufficiently similar to suggest the validity of the findings of this study.

The cotton workers’ study was started in the mid-1960s and consequently benefits from very long follow-up. However, key confounding variables such as lipid levels, diabetes, weight, body mass index and blood pressure were not included because their role in longevity was not understood at the time the study was designed and this is a limitation of the analysis. Also, smoking consumption in the cotton workers’ study was self-reported, similar to many studies of this type. However for the cotton workers’ cohort self-reporting was in 1966 to 1970, a period of time when smoking in the UK had wide social acceptance. Participants were recording current behaviour and hence bias in reporting would be small. However, no information was collected regarding smoking consumption for the participants during the many years of follow-up and only subsequent embarkation or cause of death was recorded. This presents a limitation to the interpretation of the study findings. If, for example, women participants had found smoking cessation more difficult to achieve than their male counterparts, this could provide an alternative explanation for their greater susceptibility to the detrimental effects of smoking due to greater total lifetime exposure. Whether women in the cohort would have found smoking cessation more difficult than men is difficult to assess. A comparison of data from three general population surveys conducted in 2006 to 2007 in the USA, Canada and Britain concluded that, across all age groups, there was little difference in cessation between the sexes [33]. However, a comparison of two case–control studies for lung cancer, one completed in 1950 and one completed in 1990 showed that, amongst the controls, prevalence of smoking had fallen from 79.2% to 21.5% for men and from 38.0% to 20.1% for women over those four decades [34]. The study concluded that women and older men were more likely to have been persistent cigarette smokers throughout their lives compared to men in early to middle age. In conclusion therefore, there was a statistically significant gender difference in relative long term survival comparing light smokers at baseline. The observed gender difference could be due to differences in physiology between men and women. However, an alternative explanation might be gender based differences in smoking cessation and this possibility cannot be discounted.

The finding, of a dose response relationship in which light smoking at baseline showed a greater gender differential compared to medium and heaving smoking, was a surprising outcome. The data available in this study cannot provide an explanation of this outcome but it does suggest that future studies should not compare smokers with non-smokers but that gender comparisons should be stratified by level of tobacco consumption.

## Conclusions

The results of this study demonstrate a dose response relationship in the relative risk ratios of women to men smoker to never smoked. The finding of a dose response relationship and a failure to take a relative survival approach provide an explanation for the lack of consensus in the literature about the gender difference in smoking risk. It has been noted that women smokers in the USA at least are more likely than men smokers to be light daily smokers and so the greater risk to women of light smoking will impact disproportionately on study outcomes [35].

## Competing interests

The author declare that she have no competing interests.

## Authors’ information

MAH holds a PhD by published work is a chartered statistician with the Royal Statistical Society and is a Senior Lecturer in Medical Statistics at the University of Central Lancashire, UK.

## Pre-publication history

The pre-publication history for this paper can be accessed here:

http://www.biomedcentral.com/1471-2458/14/95/prepub
